# Disposable Microchip Platform with Removable Actuators Using SAW Excitation

**DOI:** 10.1021/acsmeasuresciau.5c00027

**Published:** 2025-07-27

**Authors:** Akinobu Yamaguchi, Masatoshi Takahashi, Satoshi Amaya, Tsunemasa Saiki

**Affiliations:** † Department of Electrical, Electronic and Communications Engineering, Faculty of Science and Engineering, Toyo University, 2100 Kujirai, Kawagoe, Saitama 350-8585, Japan; ‡ Laboratory of Advanced Science and Technology for Industry, 12744 University of Hyogo, 3-1-2 Kouto, Kamigori, Ako-gun, Hyogo 678-1205, Japan; § Department of Mechanical Engineering, Graduate School of Engineering, 13143 The University of Tokyo, 7-3-1, Hongo, Bunkyo-ku, Tokyo 113-8656, Japan; ∥ Manufacturing Technology Department, Hyogo Prefectural Institute of Technology, 3-1-12, Yukihira, Suma, Kobe, Hyogo 654-0037, Japan; ⊥ Faculty of Informatics, 539877 The University of Fukuchiyama, 3370 Hori, Fukuchiyama-city, Kyoto 620-0886, Japan

**Keywords:** surface acoustic wave, microactuator, microstirrer, disposable, droplet, powder, ELISA

## Abstract

A surface-acoustic-wave-driven microactuator that allows separation of the piezoelectric substrate and chip has been fabricated and characterized. By simply placing the microactuator on a disposable chip, the microactuator did not contaminate the substrate with any reagent and could easily transport droplets and powders. The microactuator also allowed mixing of heterophase materials, such as powder and droplets, in a microfluidic well to increase their chemical reaction. This microactuator will enable significant cost savings and automation of plants and research facilities.

## Graphical abstract

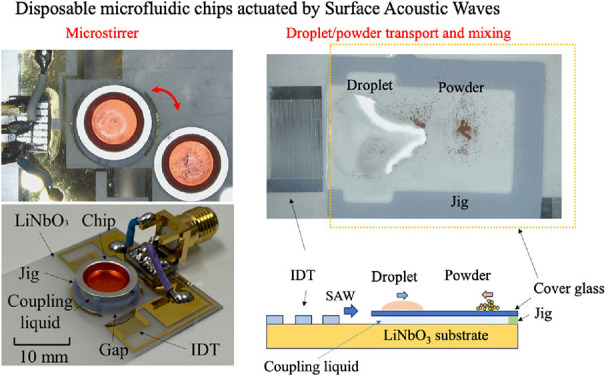

## Introduction

In recent years, micro chemical analysis systems (micro total analysis systems, μTAS) have attracted interest in the fields of medicine, environmental measurement, and biochemistry.^1–6^ μTASs have the following advantages: (1) measurement of small sample volumes, (2) short reaction times, (3) automation of measurements, including pretreatment, (4) downsizing of equipment, (5) disposability of the equipment, and (6) low cost and reduced manpower. In particular, a μTAS, which is also known as a lab-on-a-chip (LOC), is expected to integrate all of the chemical and biochemical analysis operations, such as sample preparation, reaction, separation, and detection, performed in laboratories and testing centers, on a single chip to further increase the processing capacity. Therefore, in order to integrate multiple small devices such as pumps, valves, and sensors, on a single chip, it is desirable that the manufacturing process for each device is simple.

The micropumps commonly used in chemical processes include mechanical diaphragm pumps and nonmechanical electro-osmotic pumps. Diaphragm pumps pump liquid by causing displacement of the channel volume by elastic deformation. However, diaphragm pumps have a complex structure. Electro-osmotic pumps use electro-osmotic flow.^7,8^ Electro-osmotic flow is the phenomenon where positive and negative charges are separated and face each other at the contact surface of a solid and a liquid. When an electric field is applied along the flow path, the ions in the liquid are transported according to the movement of the electric field. However, in order to pump a liquid using electroosmotic flow, the solution must be an electrolyte.

Although the example of a pump is described here, a mechanical actuator mechanism, including a pump, is essential to drive the actual microchip, which needs to pump continuous fluids and transport droplets and powders. Syringe pumps are often used in microchemical systems that are fabricated and evaluated in the laboratory for research purposes. Syringe pumps have excellent controllability, but the entire system is connected to tubing and a syringe pump, which increases the size and is not suitable for in situ measurements in an external environment, e.g., for environmental analysis. In addition, residual fluid remains in the tubing and inside the syringe, making it unsuitable for ultratrace measurements, and other mechanisms have been requested.

In 1885, Lord Rayleigh reported on surface acoustic waves (SAWs), which propagate by concentrating energy only on the surface of an elastic body.^9^ Since the discovery of SAWs, physicists have performed extensive fundamental research on SAWs in relation to seismic waves, and methods for analyzing SAWs have been established. In 1965, the generation and detection of SAWs became remarkably efficient owing to the development of an interdigital transducer (IDT) by Bell Laboratories.^10^ This, together with the discovery of lithium niobate^11–13^ and lithium tantalate,^14,15^ which have large piezoelectric effects, led to the generation of SAWs with high efficiency. The SAWs have been actively investigated for practical applications over the last 30 or so years. For example, in the field of wireless communications, noise-reduced data transmission is achieved owing to the SAW bandpass filters. Today, the wireless communication environment would be unthinkable without SAW bandpass filters. Currently, SAW bandpass filters are used for mobile phones and other applications are in practical use, but actuators^15–18^ and sensors^19–21^ using SAWs are still in the research stage.^22^

The IDTs used to generate SAWs have a very simple structure and are easy to fabricate, making them suitable for microsystems, such as μTASs. Various applications of SAW devices have been reported, such as fluid drive,^22^ droplet transfer,^17,23,24^ continuous fluid pumping on a single chip,^25^ and mixing.^25,26^ However, depending on the fluid used, the substrate can become contaminated and cannot be reused, because the microfluidic channels are directly formed on the piezoelectric substrate and the liquid is directly manipulated on the substrate. Hence, there has been a demand for the possibility of separating the microchemical system containing the driving mechanism and the microfluidic channels by SAWs. Kondoh and co-workers^27^ and Yamaguchi et al.^28^ responded to this demand by showing that the SAW-excited piezoelectric substrate and the microchannel can be separated by SAW propagation through the liquid. Since then, continuous fluid pumping^29^ and cell sorting^30–36^ have also been investigated in systems that allow separation of the chip from the piezoelectric substrate.^27,28,37–40^ As mentioned above, various examples have been reported, but none have been utilized for powder transport or droplet/powder mixing. In μTAS, many analyses and other operations are carried out by chemical operations using liquids and droplets, but unit chemical operations such as dissolving powders into liquids or extracting components from liquids to obtain them as powders for transport are also essential for automating the system.

In this study, a SAW device capable of separating the microchip from the piezoelectric substrate, serving as the drive source, was fabricated. The SAW device was fabricated by propagating the longitudinal waves of the SAWs generated by an IDT through the liquid on the substrate to drive the liquid on the microchip.^27,28,37–40^ Regardless of the reagents used, contamination of the piezoelectric substrate can be prevented by simple replacing the chip, and the SAW device can be repeatedly reused. In addition, the dead volume can be significantly reduced, and pump-less and valve-less microsystems can be fabricated. Three example applications of the SAW-driven microfluidic device are reported: acceleration of the antigen alternation reaction by introducing a mixing process, droplet transport, and powder transport are simultaneous transport and mixing of liquids and powders.

## Design Concept and Features

The design consideration for disposability is the physical separation between the piezoelectric substrate (SAW actuator) and the microchip (reaction or transport site). The disposable chip, made of a low-cost, easily replaceable material such as an aluminum plate or cover glass, is placed above a coupling liquid, allowing efficient SAW energy transmission without direct contact. A custom-designed 3D-printed jig ensures precise alignment, prevents coupling liquid dispersion, and enables repeated use of the actuator without contamination. This standardized disposable interface minimizes cross-contamination of reagents and facilitates integration into automated screening systems and factory or laboratory automation environments.

A medium (coupling liquid) is needed between the microchip and the SAW actuator to propagate the mechanical vibrations caused by the SAW. However, if the medium is confined, it becomes difficult to propagate the SAWs through the medium to the microchip. Therefore, it is necessary to consider a structure in which the microchip is loosely fixed to the substrate, i.e., to the extent that it is fixed by static frictional forces, and the SAWs can propagate through the medium. Therefore, as shown in Figure 1a–c, a 3D printer is used to form a jig with a structure in which an aluminum (Al) disposable microchip placed on the micromixing machine and connected to the piezo substrate with a coupling fluid. The design policy is to coexist a structure where the substrate and the jig are in contact with each other, such that the jig is fixed and the SAW directly propagates mechanical vibrations to the medium, as shown in the Figure 1b,c. A circular jig structure is prepared, with a pair of areas in contact with the substrate and a pair of areas not in contact with the substrate, which are formed in opposite directions; by placing the areas not in contact with the substrate in the direction of SAW propagation, SAW propagation is transmitted to the medium, enabling SAW propagation to an Al microchip placed on the jig.

**Figure 1:**
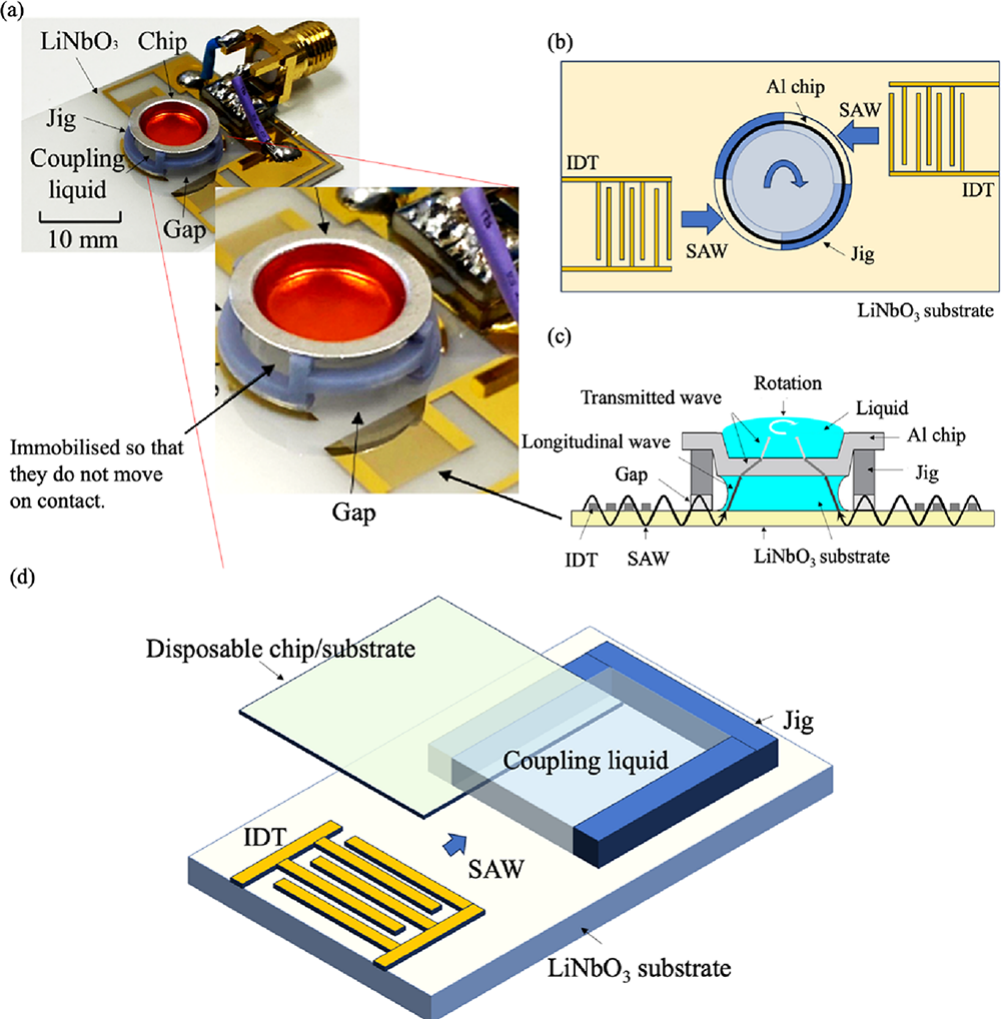
(a) Photograph of the micromixing system and magnified view of the jig section. Schematic view of the micromixing system from the (b) top and (c) cross-section of the micromixer. (d) Schematic view and layout of the disposable microchip platform with one IDT.

For microchips transporting droplets or powders, the same design guidelines should be used to fabricate the structure: in the case of a single IDT, the jig should be fabricated in the area of SAW propagation where it does not contact the substrate, as shown in Figure 1d, so as not to interfere with SAW propagation.

## Materials and Device Fabrication

The fabrication procedure of disposable microstirrer is shown in Figure 2. The device was mainly fabricated by the lithography and etching techniques used in the normal semiconductor manufacturing process.^28,37–39^ A piezoelectric substrate (LiNbO_3_: 128° rotating Y-plate X propagation) was used as the starting substrate to induce the SAWs. The thickness of the piezoelectric substrate was 500 μm, and its surface roughness was less than 0.3 nm. Cr and Au were sequentially deposited on this piezoelectric LiNbO_3_ substrate by radio frequency (RF) sputtering (CFS-4EP-LL, Shibaura Mechatronics). The thicknesses of the Cr and Au films were 10 and 100 nm, respectively. Next, ultraviolet (UV) exposure was performed using a positive resist (OFPR800LB-20 cP, Tokyo Ohka Kogyo). The IDT pattern on the glass mask was transferred using a mask aligner (MA6, SUSS Micro Tec). Based on this pattern, etching of Au and Cr was then performed, and the resist was peeled off to fabricate the IDT (pitch = 200 μm, aperture width = 6.0 mm, log 20), which served as the SAW generator.

**Figure 2:**
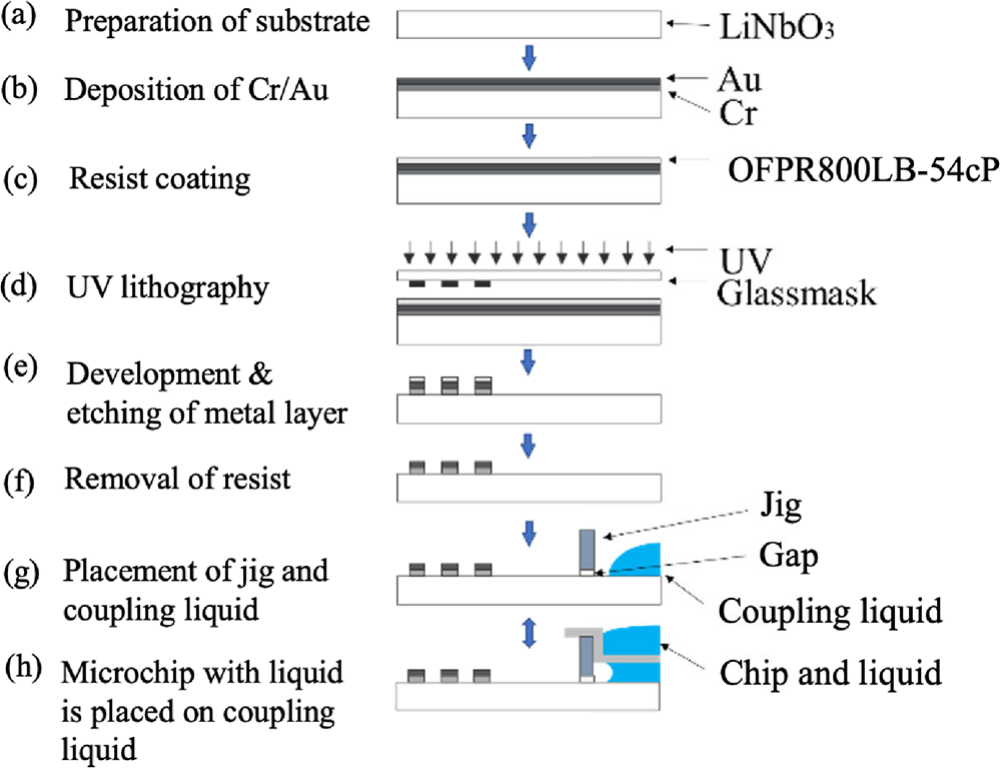
Schematic diagram of the device and system fabrication process. (a) Preparation of the LiNbO_3_ substrate as the starting substrate. (b) Deposition of Cr/Au onto the substrate by a magnetron sputtering. (c) Resist coating for ultraviolet (UV) lithography. (d) UV lithography for patterning the interdigital transducer (IDT) structure. (e) Development and metal etching. (f) Removing the resist. (g) Placement of the jig and coupling liquid. (h) Placement of the microchip with liquid on the coupling liquid.

The SAW-induced microstirrer system was then composed of placing a jig made with an optical three-dimensional (3D) printer (Mars 2 Pro by Elegoo) and placing an Al chip (outer diameter = 10 mm, height = 2.1 mm, thickness = 0.1 mm, density = 2.7 g/cm^3^, B0143017, PerkinElmer) on the LiNbO_3_ piezoelectric substrate. The distance from the substrate to the Al chip was determined by adjusting the height of the jig. The jig was designed so that the Al chip did not directly touch the surface of the LiNbO_3_ substrate, which was the incident area.

Both cover glass and Al chips are available cheaply in large quantities and are suitable for applications such as environmental analysis and medical testing, where unit chemical operations should be carried out without contamination.

## Experimental Section/Methods

This paper presents the results of four experiments, with a discussion of each experimental topic. Details of the experiments can be found in the ‘Results and Discussion’ section for each topic. This section described the common device handling and experimental methods used in the experiments.

For SAW excitation, the IDT is basically powered under the following experimental conditions: The RF voltage supplied to the IDT was a 1 kHz burst voltage consisting of 2000 cycles of a 19.12 MHz sine wave generated by a signal generator (AFG3252, Tektronix) and amplified by a RF amplifier (ALM00110-2840FM, R&K). The power supplied to the IDT was regulated by the voltage of the signal generator and checked with a RF power meter (NRP-Z91 and AR, DC3001M1, Rohde & Schwarz). The peak-to-peak output voltage of the arbitrary waveform signal generator was set arbitrarily from 0 to 5 V.

In the case of disposable micromixing systems, opposing IDTs were electrically connected and electric power was applied under the above conditions in the same phase. The jig should be constructed with a gap in the area where the SAW is propagated into the coupling liquid, and the rest of the jig should be installed on the LiNbO_3_ substrate, as shown in Figures 1b and 2g. An Al chip was placed on top of the jig for the experiment. For droplet and powder transport, a cover glass, commonly used in microscopy, was used as a disposable microchip. A gap was left open between the cover glass and the LiNbO_3_ substrate in the area where the jig was placed to propagate the SAW into the coupling liquid, while the other areas were directly contacted to the LiNbO_3_ substrate to prevent the cover glass and jig from moving. In this transport experiment, a device with a single IDT was used for simplicity; the SAW excitation conditions were the same settings as those described above. All experiments were performed at room temperature and in air.

## Results and Discussion

### Disposable Microstirrer for Enzyme-Linked Immunosorbent Assay (ELISA)

A disposable SAW-driven microstirrer was used to react the enzyme-labeled antibodies and substrates used in enzyme-linked immunosorbent assay (ELISA). The typical ELISA procedure is shown in Figure 3a. In this experiment, procedure 6 in Figure 3a was compared with and without the use of the microstirrer. Anti-IgG­(H+L), Mouse, Goat-Poly, horseradish pyruvate oxidase (HRP) (1 mg/mL) was used as the enzyme-labeled antibody, and the tetramethylbenzidine (TMB) Microwell Peroxidase Substrate System (two-component system) was used as the substrate solution.

**Figure 3:**
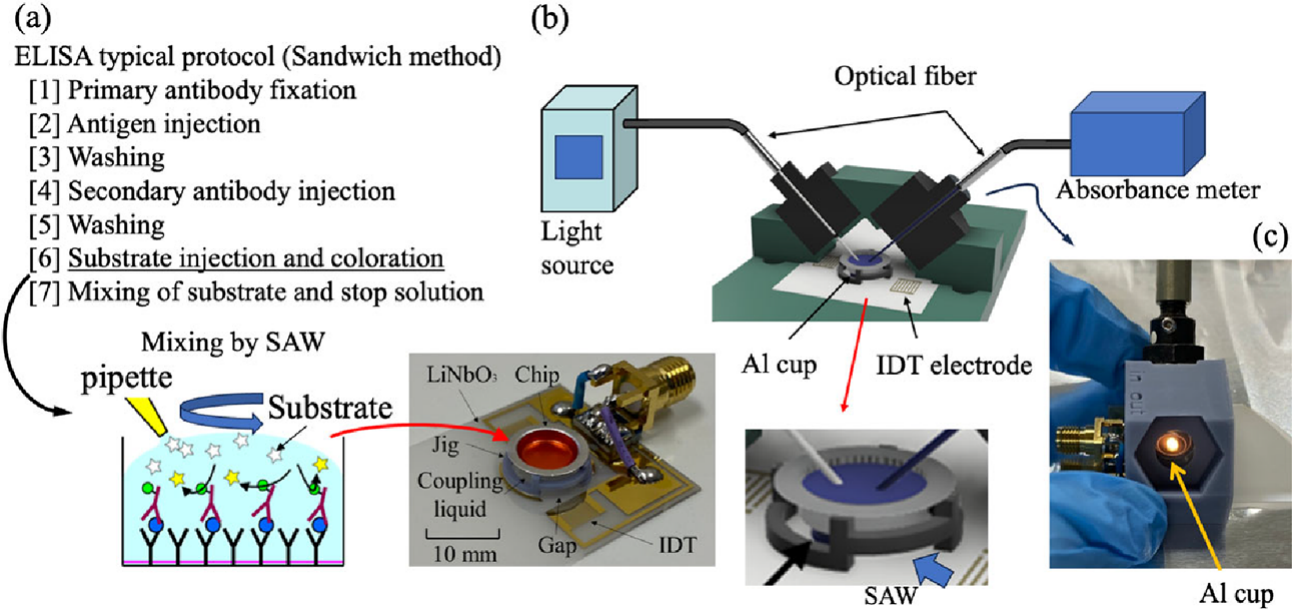
(a) Typical protocol of enzyme-linked immunosorbent assay (ELISA) for the sandwich method. Schematic diagram and photograph of the substrate injection and coloration reaction (procedure 6) in the micromixer with surface acoustic waves (SAWs). (b) Schematic diagram of the system for measuring the absorbance during micromixing. The light generated by the light source passed through an optical fiber and irradiated the microchip (Al cup). The transmitted light passed through another optical fiber, and the absorbance was measured by an absorbance meter. (c) Actual photograph of the light irradiation observed in the fabricated system.

By mixing the two solutions, the antibody-labeled enzyme HRP decomposes the hydrogen peroxide water in the substrate solution to generate the active enzyme. The produced active enzyme is highly unstable and acts as an oxidizing agent. The active enzyme oxidizes TMB, which generates an oxidized dye. The dye also absorbs light at wavelengths of 370 and 650 nm. In this experiment, the reaction rate of the enzyme substrate reaction was determined by measuring the absorbance of the reaction reagent at a wavelength of 650 nm.

In the experiment, 55 μL of the substrate solution and 5 μL of the enzyme-labeled antibody solution diluted to 100, 30, 3, 1, 0.3, or 0.1 ng/mL in phosphate-buffered saline (DPBS) were mixed on an Al chip to give a total volume of 60 μL. The experiment was performed at room temperature (20 °C). The RF voltage supplied to the IDT was a 1 kHz burst voltage consisting of 2000 cycles of a 19.12 MHz sine wave generated by a signal generator (AFG3252, Tektronix) and amplified by a RF amplifier (ALM00110–2840FM, R&K). The power supplied to the IDT was regulated by the voltage of the signal generator and checked with a RF power meter (NRP-Z91 and AR, DC3001M1, Rohde & Schwarz). As described above, the peak-to-peak output voltage of the arbitrary waveform signal generator was set arbitrarily from 0 to 5 V. The signal generated by the signal generator in burst mode was amplified by the RF amplifier and input to the SAW device, which was monitored by the RF power meter as input power rather than voltage. A RF power of 1.0 W was supplied to the IDT. When an amplified in-phase RF voltage was supplied to the IDT, SAWs were generated, which propagated across the substrate surface and reached the coupling liquid, radiating longitudinal waves. The longitudinal waves propagated to the coupling liquid, the chip, and the liquid on the chip, causing the solution to agitate. The energy efficiency of a separable (disposable) mixing system with coupling liquid is about 88% for distance of 1.0 mm between SAW substrate and chip, as described in the Supporting Information (Figure S1). The energy efficiency of a separable mixing system is considered to be sufficiently high to be worthwhile considering the pollution problems and the cost of fabricating SAW devices. The distance from the substrate to the chip was 1.0 mm, and 40 μL of pure water was used as the coupling liquid. Here, when power of 1.0 W was supplied to the IDT, the solution temperature increased to 30 °C. Therefore, for comparison, in the experiment without stirring, the experiment was carried out at 30 °C on a hot plate.

A schematic diagram of the detection system is shown in Figure 3b. First, the light emitted from a halogen light source (DH-2000) passes through an optical fiber and collimating lens and is incident on the solution on the Al chip. The transmitted light that passes through the solution and is reflected at the bottom of the Al chip passes through a collimating lens and optical fiber and is incident on a spectrophotometer (HR4000), and the absorbance of the solution is measured. The jig used here was fabricated using an optical 3D printer. From a photograph of the transmitted light side shown in Figure 3c, the incident light is reflected at the bottom of the Al chip and proceeds to the spectrometer side.

The absorbance versus concentration plots for IDT power of 0 (without SAWs) and 1.0 W (with SAWs) are shown in Figure 4a. The concentration of the enzyme-labeled antibody solution is shown on the horizontal axis, and the absorbance of the reaction solution is shown on the vertical axis. The absorbance values were measured 9 min after the start of solution mixing. Compared with IDT power = 0, the reaction volume increases with agitation by SAWs (IDT power = 1.0 W) at each concentration. This is because of the increase in the number of physical collisions between the enzyme-labeled antibody and the substrate owing to agitation by SAWs. From these results, the use of a disposable SAW-driven microstirrer is expected to increase the reaction efficiency and sensitivity of enzyme reactions in ELISA. The changes of the absorbance with time for an enzyme-labeled antibody concentration of 30 ng/mL with IDT power of 0 and 1.0 W are shown in Figure 4b. The horizontal axis shows the time course from the start of solution mixing, and the vertical axis shows the absorbance. The absorbance at 9 min was 0.61 without SAWs and 1.08 with SAWs. This shows that the absorbance increased 1.8-fold with stirring by SAWs. The absorbance exceeded 0.61 after 3.5 min with SAW stirring. From these results, the reaction rate was approximately three times faster with SAW stirring than without SAW stirring. Therefore, the disposable microstirrer can be used to increase the sensitivity and speed of enzyme substrate reactions in ELISA.

**Figure 4:**
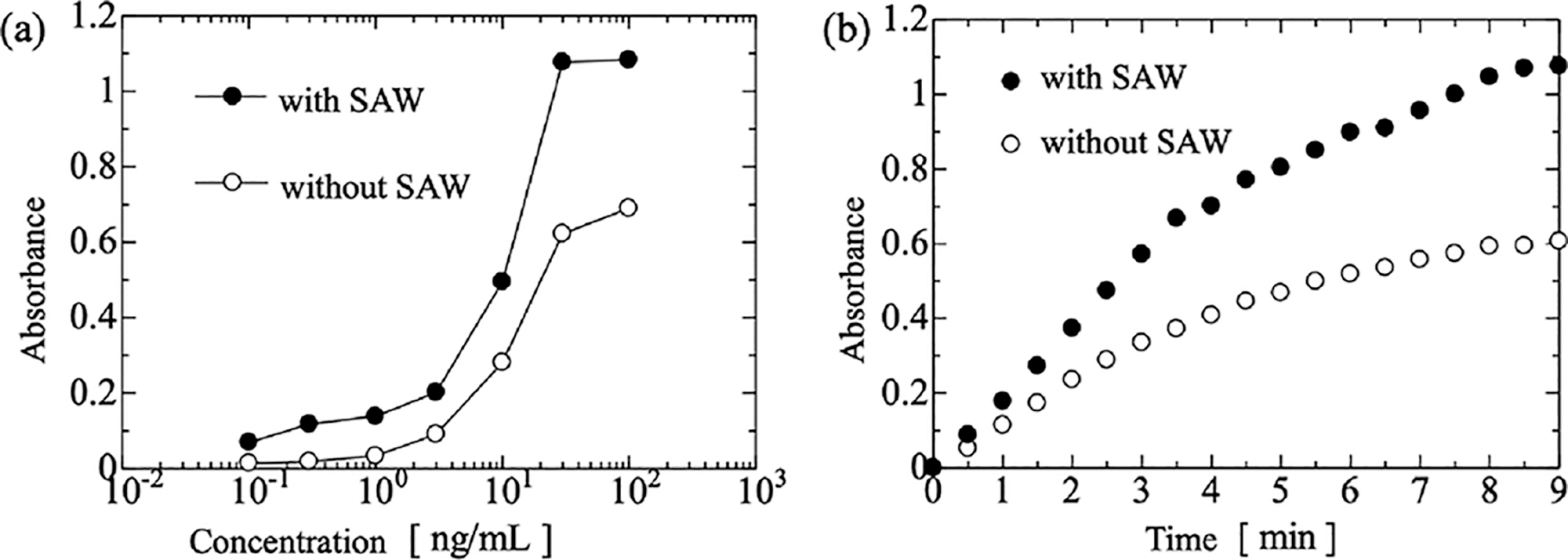
(a) Dependence of the absorbance on the concentration of enzyme-labeled antibody with and without SAW agitation. (b) Change of the absorbance with time with and without SAW agitation. The black solid and white open circles are the measured absorbances values at each concentration in the cases with and without SAW agitation, respectively.

### SAW-Driven Droplet Transport on a Disposable Microchip

The devices used in the droplet transport experiments were fabricated by the same technique as that described above. A photograph of the disposable microactuator is shown in Figure 5a, and a schematic of the cross-section of the device is shown in Figure 5b. SAWs generated by the IDT (pitch = 200 μm, aperture width = 6.0 mm, log 20) and propagating on the piezoelectric substrate surface were incident on the coupling liquid. The SAWs then reached the cover glass placed on the 0.5 mm-high jig and propagated to the droplet on the cover glass. To reduce the friction at the solid/liquid interface on the cover glass, a fluorine coating material (Cytop) was deposited (150 nm thick) on the cover glass.^41^ The contact angle of the untreated cover glass was 20°, whereas that on the Cytop coating was 112°.

**Figure 5:**
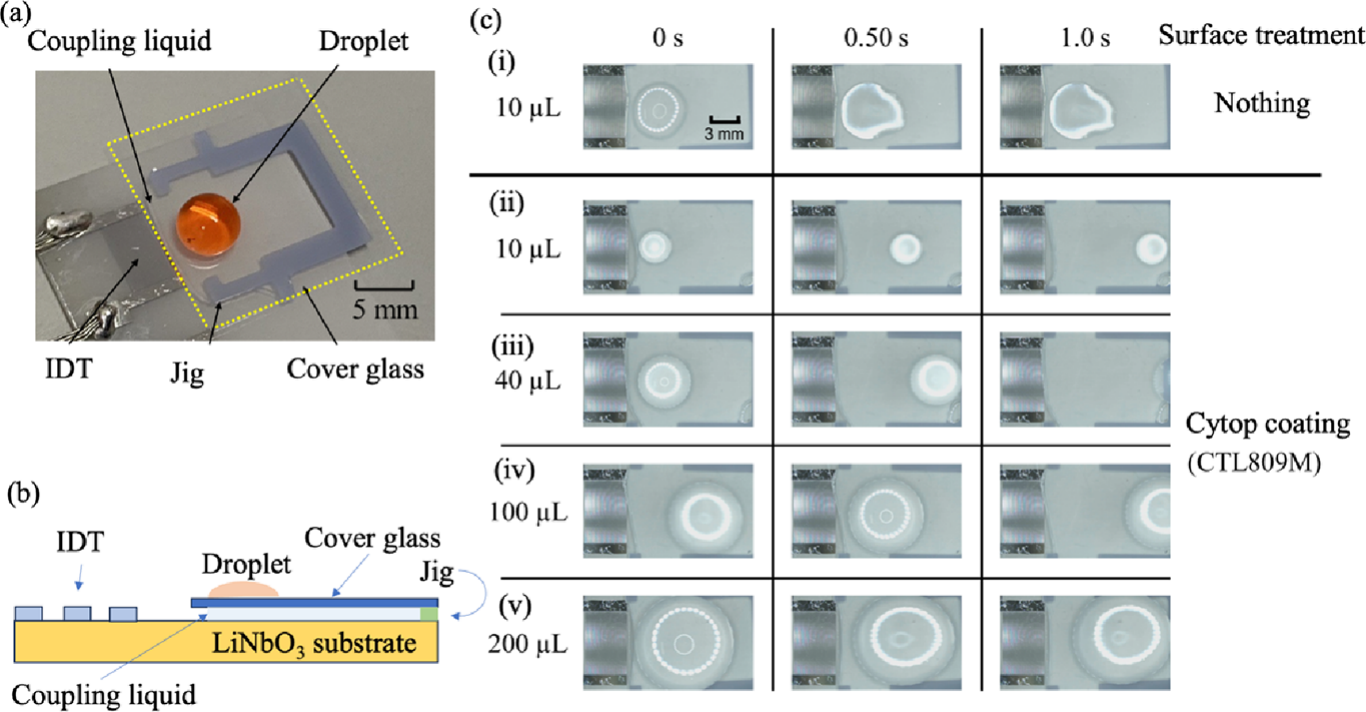
(a) Optical photograph of the entire system. The area enclosed by the yellow dotted line is the cover glass, which was placed on top of the coupling liquid and jig on the piezoelectric substrate. (b) Schematic diagram of the cross-sectional structure of the entire system. (c) (i) Photographs of a 10 μL droplet dropped driven by SAWs on the cover glass surface without any surface treatment at 0, 0.5, and 1.0 s. Photographs of (ii) 10 μL, (iii) 40 μL, (iv) 100 μL, and (v) 200 μL droplets dropped and driven by SAWs on Cytop-coated cover glass.

The transport velocities of 1.0, 2.0, 3.0, 4.0, 5.0, 10, 20, 30, 40, 50, 60, 70, 80, 100, 150, 200, and 250 μL droplets of pure water on a cover glass were measured with IDT power of 1.0 W. A typical movie for the transport a 10 μL droplet on Cytop-coated cover glass is shown in the Supporting Information Movie No. 1. Images of the transport of a 10 μL droplets on untreated cover glass and 10, 40, 100, and 200 μL droplets on Cytop-coated cover glass are shown in Figure 5c. Comparing with the results of droplet transport on untreated cover glass and Cytop-coated cover glass, the Cytop-coating dramatically improved the transport properties owing to the change in the contact angle. The droplet volume dependence of the droplet transport velocity on the Cytop-coated cover glass is shown in Figure 6. The transport velocity of droplets with volume less than 10 μL were not stable. This is considered to be due to variation in the wettability of the Cytop surface. The dependence of the transport velocity of the droplet on the variation of the wettability on the cover glass increased with decreasing droplet size, and the droplet sometimes stopped or slowed down during droplet transport. The transport of the 40 μL droplets was the most stable and fastest. Droplets with volumes above 100 μL could also be transported, although they were larger than the aperture width of the IDT. The ability of this disposable micropump to transport droplets of 10 ∼ 200 μL droplets shows the potential of the micropump for droplet transport.

**Figure 6:**
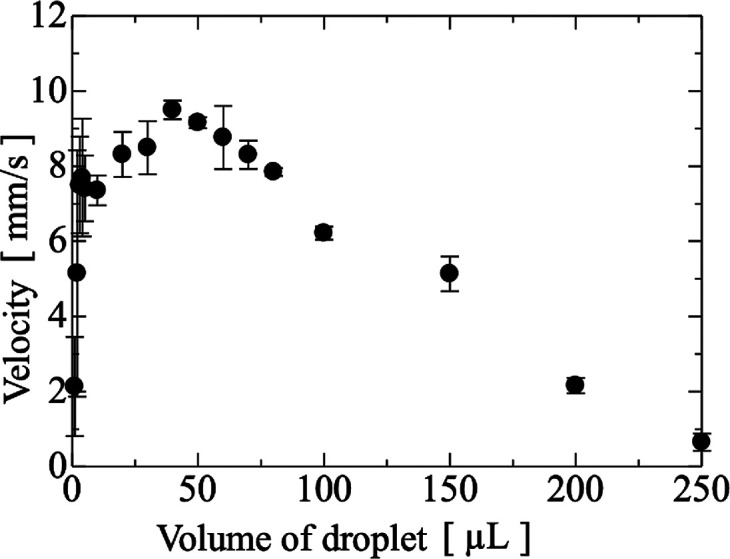
Droplet volume dependence of the droplet velocity.

### SAW-Driven Powder Transport on a Disposable Microchip

Images of the transport of copper powder (40 μm particle diameter) on a cover glass coupled through the coupling liquid on the piezoelectric substrate are shown in Figure 7. Images of the copper powder at 0 s and 10, 20, 30, 60, 120, and 180 s from the start of power 1.0 W supply to the IDT are shown in Figure 7a–g, respectively. The powder was transported in the opposite direction to the SAWs generated by the IDT. This suggests that Rayleigh waves were also generated on the cover glass surface. This result is consistent with the previous studies.^42,43^ However, this is the case when cover glass was used, and it depends on the material properties of the chip used. Many variables of the powder should be considered, such as the atomic structure of the material, the interatomic distances, and the atomic interactions of the powder. Future investigations should be carried out using network analyzers and laser Doppler meters to elucidate the principles of the waves generated on the chip surface.

**Figure 7:**
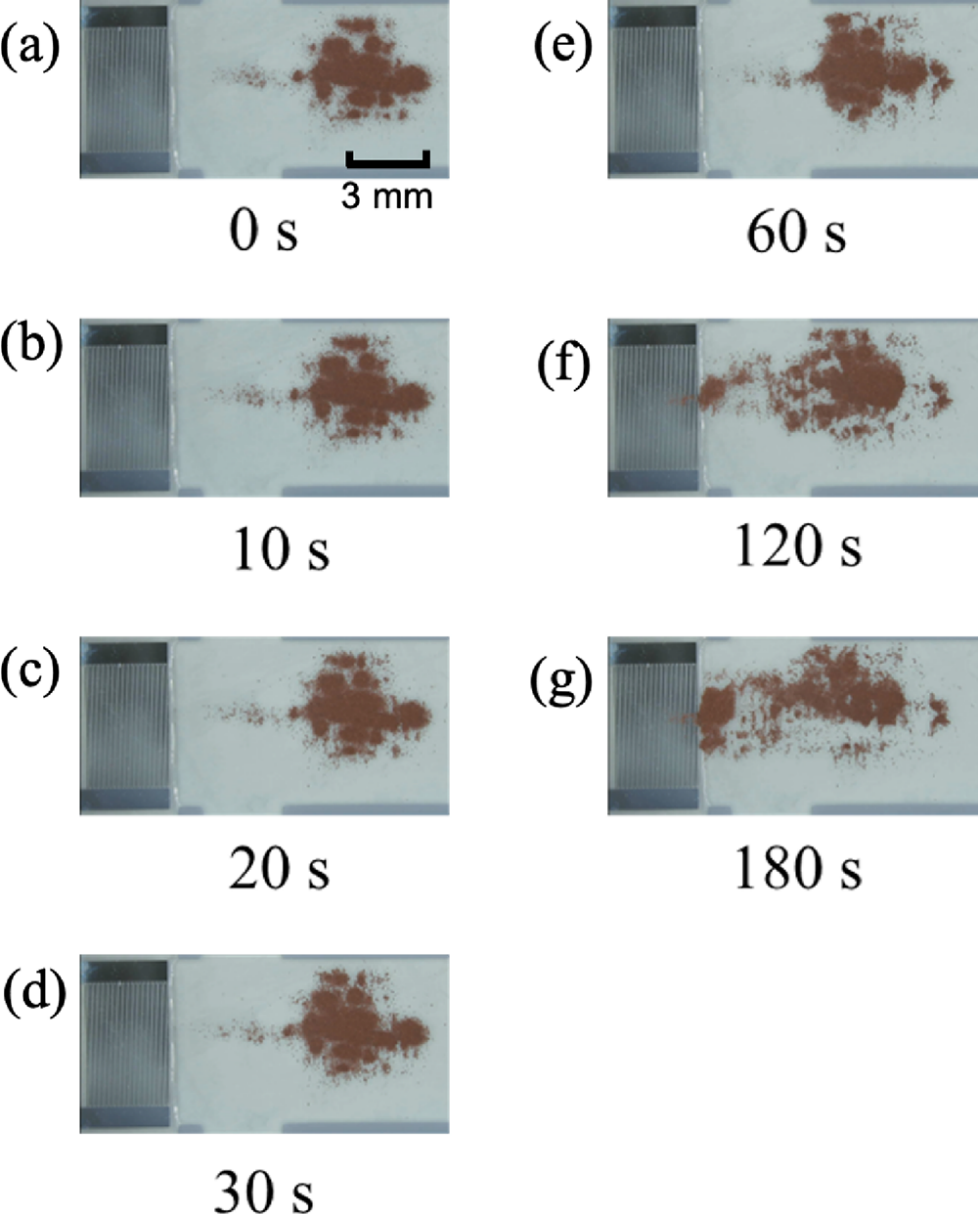
Photographs of SAW-driven transport of copper powder (particle size of 40 μm) on cover glass at (a) 0, (b) 10, (c) 20, (d) 30, (e) 60, (f) 120, and (g) 180 s.

The powder transport characteristics of the powder transport on the cover glass connected by a coupling liquid and directly on the piezoelectric substrate were compared. Cross-sectional schematic diagrams of the powder transport directly driven on the piezoelectric substrate and on the cover glass are shown in Figure 8a,b respectively. The actual photographs of both cases are shown in Figure 8c,d. In both cases, the powder was transported to the IDT side, and it was therefore transported by backward elliptical vibrations excited by the piezoelectric substrate, as schematically illustrated in Figure 8a,b. Comparison of the photographs in Figure 8c,d showed that the wavelengths of the standing waves were approximately the same. However, when the powder was transported on the cover glass (Figure 8c), all of the powder horizontally spread, indicating that the vibration amplitude from the piezoelectric material was weakened and the effective full width at half-maximum (fwhm) increased. Typical example movies for the powder transport on LiNbO_3_ substrate and on cover glass coupled through the coupling liquid (distance 1.0 mm) LiNbO_3_ substrate are shown in the Supporting Information Movies No. 2 and No. 3, respectively.

**Figure 8:**
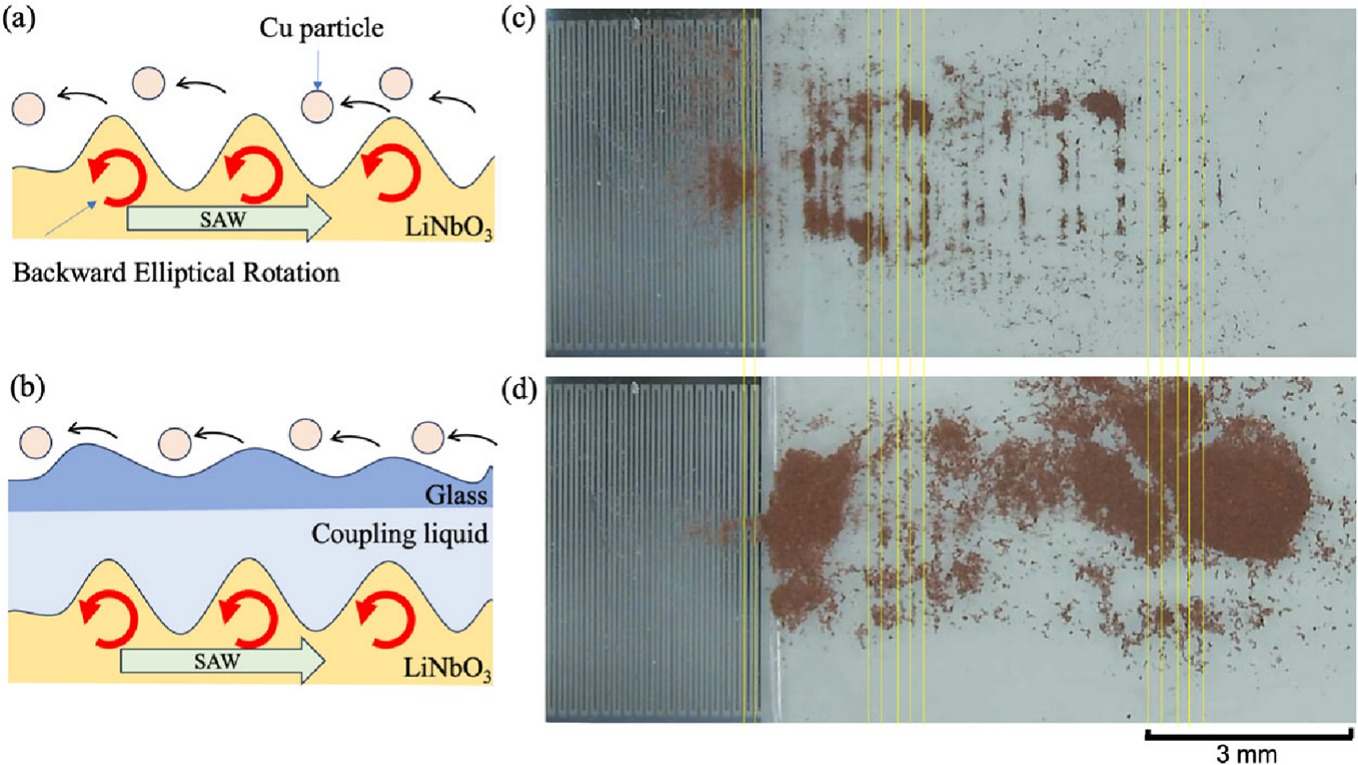
Cross-sectional schematic diagrams of SAW-driven powder transport (a) directly on the piezoelectric substrate (LiNbO_3_) and (b) on the cover glass coupled with the coupling liquid on the LiNbO_3_ substrate. Photographs of the SAW-driven powder transport experiments on (c) the piezoelectric substrate and (d) cover glass at arbitrary time. In both cases, standing waves were induced. The yellow lines are included to compare the respective periods.

As discussed above, the vibration amplitude from the SAWs is weakened in the disposable microsystem, but that powder transport is also possible without contamination. To improve the propagation characteristics of the SAWs, impedance matching can be achieved by changing the coupling liquid and materials of the microchip.

### SAW-Driven Simultaneous Powder and Droplet Handling and Mixing

We have shown that it is possible to transport droplets and powders using the disposable microchips. We then thought that it might be possible to simultaneously transport and mix liquids and powders using the SAW-driven microdevice. As an example, both a droplet and a powder were placed on the cover glass which was coupled through the coupling liquid on the LiNbO_3_ piezoelectric substrate, as shown in Figure 9a. The time course observations after the application of the SAWs are shown in Figure 9b–f. The directions of the propagation of the liquid and powder were opposite, which means that they collided and the powder was encapsulated in the droplet. From the time course observations, the powder exhibited swirling behavior within the droplet. After 25 s (Figure 9f), a large vortex structure formed within the droplet. These results show that the disposable system is capable of simultaneously performing unit operations on powders and droplets (see the Supporting Information, Movie No. 4). Combining this SAW-based micropump enables the microfluidic device to be fully disposable as shown in the Supporting Information Movie No. 5.

**Figure 9:**
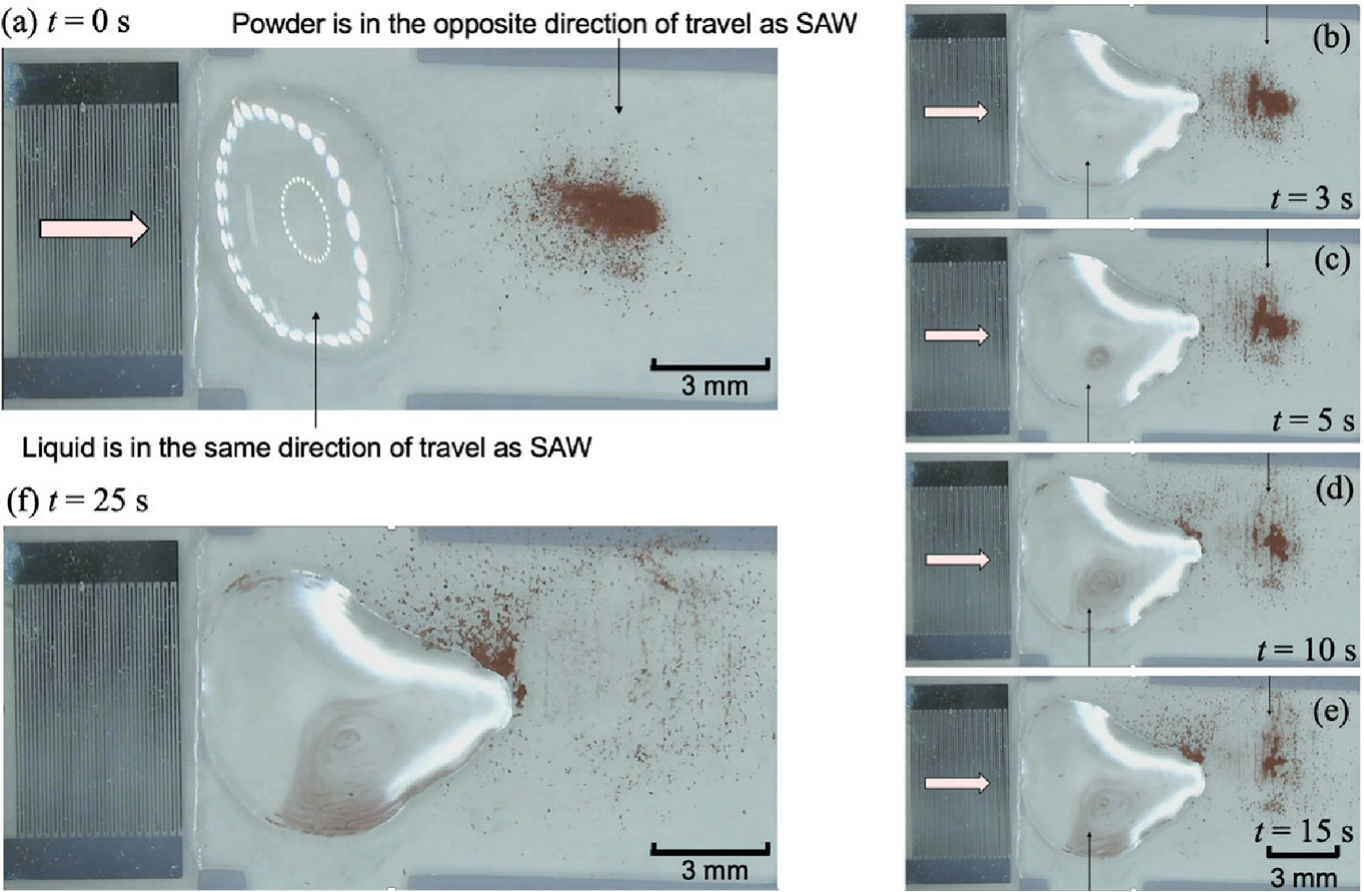
(a) Photographs of the simultaneous transport and mixing of a droplet and copper powder on the cover glass. The SAWs propagated from the IDT in the direction of the arrow. The droplet was placed closer to the IDT and the powder was placed farther away. (b–f) Photographs observed after 3, 5, 10, 15, and 25 s, respectively.

## Conclusions

We have reported three applications of SAW-driven disposable microsystems. First, using a SAW-driven disposable microstirrer for enzyme substrate reactions in ELISA increased the reaction efficiency and sensitivity. Second, a SAW-driven disposable micropump showed the ability to transport droplets with volumes ranging from 10 to 200 μL. Third, transport of copper powder (40 μm particle size) was successfully achieved with a SAW-driven disposable microdevices. The powder was transported in the opposite direction of the SAWs generated by the IDT. As an example of an interesting applications of the microdevice, SAW-driven simultaneous powder and droplet handling and mixing was demonstrated.

In the future, the disposable microfluidic platforms actuated by SAW will be applied to flow microsystems for continuous liquid manipulation to further expand their applications. This microfluidic platform ensures that the reagents do not contaminate the substrate, irrespective of the types of reagents, and that the microfluidic platform can be easily manipulated by simply replacing the chip. In addition, this microfluidic platform will enable significant cost savings and automation of factories and research facilities.

## Supplementary Material













## Notes

Movie 1 (MP4)
Movie 2 (MP4)
Movie 3 (MP4)
Movie 4 (MP4)
Movie 5 (MP4)
Energy efficiency evaluation and experimental videos of the disposable system with coupling liquid as the medium (PDF)

## References

[1] Culbertson C. T., Mickleburgh T. G., Stewart-James S. A., Sellens K. A., Pressnall M. (2014). Micro Total
Analysis Systems: Fundamental Advances and Biological Applications. Anal. Chem..

[2] Reyes D. R., Iossifidis D., Auroux P.-A., Manz A. (2002). Micro Total Analysis
Systems. 1. Introduction, Theory, and Technology. Anal. Chem..

[3] Auroux P.-A., Iossifidis D., Reyes D. R., Manz A. (2002). Micro Total Analysis
Systems. 2. Analytical Standard Operations and Applications. Anal. Chem..

[4] Sajeesh P., Sen A. K. (2014). Particle separation
and sorting in microfluidic devices:
a review. Microfluidics Nanofluidics.

[5] Shields C. W., Reyes C. D., López G. P. (2015). Microfluidic
cell sorting: a review of the advances in the separation of cells
from debulking of rare cell isolation. Lab Chip.

[6] Contreras-Naranjo J. C., Wu H.-J., Ugaz V. M. (2017). Microfluidics for exosome isolation
and analysis: enabling liquid biopsy for personalized medicine. Lab Chip.

[7] Wang X., Cheng C., Wang S., Liu S. (2009). Electroosmotic pumps
and their applications in microfluidic systems. Microfluid Nanofluid.

[8] Gao M., Gui L. (2014). A handy liquid metal
based electroosmotic flow pump. Lab Chip.

[9] Rayleigh L. (1885). On waves propagated
along the plane surface of an elastic solid. Proc. London Math. Soc..

[10] Sitting, E. K. Elastic surface wave device. US Patent 3,360,749 (Dec. 9 1964).

[11] Renaudin A., Chuda K., Zhang V., Coqueret X., Camart J.-C., Tabourier P., Druon C. (2005). Saw lab-on-chip in
view of protein
affinity purification implemented from nanodroplet transport. Proc. μTAS 2005.

[12] Sritharan K., Strobl C. J., Schneider M. F., Wixforth A., Guttenberg Z. (2004). Acoustic mixing
at low Reynold’s numbers. Appl. Phys.
Lett..

[13] Akio Y., Masahiro N., Nobuhiro T., Toshiro H. (2005). An integrated droplet
manipulation device using surface acoustic wave. Proc. μTAS 2005.

[14] Hashimoto, K. ; Yamaguchi, M. ; Mineyoshi, S. ; Kawachi, O. ; Ueda, M. ; Endoh, G. ; Ikata, O. Optimum leaky-SAW cut of LiTaO3 for minimised insertion loss devices. In Proc. 1997 IEEE Ultrason. Symp.; IEEE: 1997, pp 245–254.

[15] Koskela J., Knuuttila J. V., Makkonen T., Plessky V. P., Salomaa M. M. (2001). Acoustic
Loss Mechanisms in Leaky SAW Resonators on Lithium Tantalate. IEEE Trans. Ultrasonics, Ferroelectrics, and frequency control.

[16] Kurosawa M., Takahashi M., Higuchi T. (1996). Ultrasonic Linear Motor Using Surface
Acoustic Waves. IEEE Trans. Ultrason. Ferroelectr.
Freq. Control.

[17] Wixforth A. (2003). Acoustically
driven planar microfluidics. Superlattices Microstruct..

[18] Chono K., Shimizu N., Matsui Y., Kondoh J., Shiokawa S. (2004). Development
of Novel Atomization System Based on SAW Streaming. Jpn. J. Appl. Phys..

[19] Andle J. C., Vetelino J. F. (1994). Acoustic wave biosensors. Sens.
Actuators A.

[20] Kondoh J., Muramatsu T., Nakanishi T., Matsui Y., Shiokawa S. (2003). Development
of practical acoustic wave liquid sensing system and its application
for measurement of Japanese tea. Sens. Actuators
B.

[21] Shiokawa S., Kondoh J. (2004). Surface Acoustic Wave
Sensors. Jpn. J. Appl. Phys..

[22] Sano A., Matsui Y., Shiokawa S. (1998). New Manipulator Based on Surface
Acoustic Wave Streaming. Jpn. J. Appl. Phys..

[23] Saiki T., Okada K., Utsumi Y. (2010). Highly efficient liquid flow actuator
operated by surface acoustic waves. IEEJ. Trans.
Electronics, Information and Systems.

[24] Tan M. K., Friend J. R., Yeo L. Y. (2009). Interfacial Jetting
Phenomena Induced
by Focused Surface Vibrations. Phys. Rev. Lett..

[25] Saiki T., Okada K., Utsumi Y. (2010). Micro liquid
rotor operated by surface-acoustic-wave. Microsyst.
Technol..

[26] Sritharan K., Strobl C. J., Schneider M. F., Wixforth A. (2006). Acoustic mixing at
low Reynold’s numbers. Appl. Phys. Lett..

[27] Yasuda N., Sugimoto M., Kondoh J. (2009). Novel micro-laboratory on piezoelectric
crystal. Jpn. J. Appl. Phys..

[28] Yamaguchi A., Takahashi M., Saegusa S., Utsumi Y., Saiki T. (2024). Removable
and replaceable micro-mixing system with surface acoustic wave actuators. Jpn. J. Appl. Phys..

[29] Schmid L., Wixforth A., Weitz D. A., Franke T. (2012). Novel surface acoustic
wave (SAW)-driven closed PDMS flow chamber. Microfluid. Nanofluid..

[30] Ding X., Li P., Lin S.-C. S., Stratton Z. S., Nama N., Guo F., Slotcavage D., Mao X., Shi J., Costanzo F., Huang T. J. (2013). Surface acoustic
wave microfluidics. Lab Chip.

[31] Simon G., Andrade M. A. B., Reboud J., Marques-Hueso J., Desmulliez M. P. Y., Cooper J. M., Riehle M. O., Bernassau A. L. (2017). Particle
separation by phase modulated surface acoustic waves. Biomicrofluidics.

[32] Lee J., Rhyou C., Kang B., Lee H. (2017). Continuously phase-modulated
standing surface acoustic waves for separation of particles and cells
in microfluidic channels containing multiple pressure nodes. J. Phys. D: Appl. Phys..

[33] Wu M. (2017). Isolation
of exosomes from whole blood by integrating acoustics and
microfluidics. Proc. Natl. Acad. Sci. U. S.
A..

[34] Ding X., Peng Z., Lin S-C. S., Geri M., Li S., Li P., Chen Y., Dao M., Suresh S., Huang T. J. (2014). Cell separation
using tilted-angle standing surface acoustic waves. Proc. Natl. Acad. Sci. U. S. A..

[35] Li P., Mao Z., Peng Z., Zhou L., Chen Y., Huang P.-H., Truica C. I., Drabick J. J., El-Deiry W. S., Dao M., Suresh S., Huang T. J. (2015). Acoustic separation of circulating
tumor cells. Proc. Natl. Acad. Sci. U. S. A..

[36] Lata J. P., Guo F., Guo J., Huang P-H. S., Yang J., Huang T. J. (2016). Surface
acoustic waves grant superior spatial control of cells embedded in
hydrogel bers. Adv. Mater..

[37] Terakawa Y., Kondoh J. (2020). Numerical and experimental study of acoustic wave propagation
in glass plate/water/128YX-LiNbO_3_ structure. Jpn. J. Appl. Phys..

[38] Takahashi M., Yamaguchi A., Utsumi Y., Takeo M., Amaya S., Sakamoto H., Saiki T. (2022). Micro stirrer with heater mounted
on SAW actuator for high-speed chemical reaction. J. Photopolym. Sci. Technol..

[39] Saegusa S., Saiki T., Amaya S., Fukuoka T., Takizawa Y., Amano S., Utsumi Y., Yamaguchi A. (2023). Aggregation of Au colloids using surface acoustic waves. J. Photopolym. Sci. Technol..

[40] Glass N. R., Shilton R. J., Chan P. P. Y., Friend J. R., Yeo L. Y. (2012). Miniaturized
Lab-on-a-Disc (miniLOAD). Small.

[41] Fluorochemicals developed by AGC Chemicals. https://www.agc-chemicals.com/jp/en/fluorine/products/detail/index.html?pCode=JP-EN-F019 (access verification date: May 2nd, 2025).

[42] Saiki T., Tsubosaka A., Yamaguchi A., Suzuki M., Utsumi Y. (2017). Interdigital
transducer generated surface acoustic waves suitable for powder transport. Adv. Powder Technol..

[43] Saiki T., Takizawa Y., Kaneyoshi T., Iimura K., Suzuki M., Yamaguchi A., Utsumi Y. (2021). Tranporting Poweder with Surface
Acoustic Waves Propagating on Tilted Substrate. Sens. Mater..

