# Supplementary material for: Disposable Microchip Platform with Removable Actuators Using SAW Excitation
Source: ACS Meas Sci Au. 2025 Jul 27;5(4):489–96. doi: 10.1021/acsmeasuresciau.5c00027 (PMC12371580; doi:10.1021/acsmeasuresciau.5c00027)
Supplement: Supplementary file 6 [file tg5c00027_si_006.pdf]

Supporting Information for

Disposable microchip platform with removable actuators using SAW excitation

Akinobu Yamaguchi[1*], Masatoshi Takahashi[2], Satoshi Amaya[3], Tsunemasa Saiki[4, 5]

[1]Department of Electrical, Electronic and Communications Engineering, Faculty of Science and Engineering, Toyo University, 2100 Kujirai, Kawagoe, Saitama 350-8585, Japan

[2]Laboratory of Advanced Science and Technology for Industry, University of Hyogo, 3-1-2 Kouto, Kamigori, Ako-gun, Hyogo 678-1205, Japan

[3]Department of Mechanical Engineering, Graduate School of Engineering, The University of Tokyo, 7-3-1, Hongo, Bunkyo-ku, Tokyo 113-8656, Japan

[4]Manufacturing Technology Department, Hyogo Prefectural Institute of Technology, 3-1-12, Yukihira, Suma, Kobe, Hyogo 654-0037, Japan

[5]Faculty of Informatics, The University of Fukuchiyama, Fukuchiyama, 3370 Hori, Fukuchiyama-city, Kyoto 620-0886, Japan

Contact address: yamaguchi054@toyo.jp (A.Y)*

To investigate the energy loss of the microstirrer through the coupling liquid, the results of rotating the ball on the cover glass through the coupling liquid were compared with those of a system in which the liquid reservoir was constructed directly on the $LiNbO_3$ substrate, as shown in Fig. S1. Here, case (a) was an experimental system where the liquid was placed directly on the substrate and the tracer floats in the liquid. Case (b) was an experimental system in which a coupling liquid was placed on the substrate, a cover glass was placed over the coupling liquid, a liquid reservoir was formed on the cover glass and the tracer floats in the liquid reservoir. The distance between the $LiNbO_3$ substrate and the cover glass was set to 1.0 mm. The results showed that the balls rotated at 3.2 rotation per second (rps) in case (a) and at 2.8 rps in case (b) as shown in Fig. S1. It was found that the rotational speed was 0.875 times higher in the case (b) through the coupling liquid than in the case (a) of direct, rotational rotation. If the distance is about 3 mm, this is 0.75 times greater, and although there is an energy loss, the system is considered to be sufficiently useful considering the advantages of contamination prevention and the possibility of tip replacement.

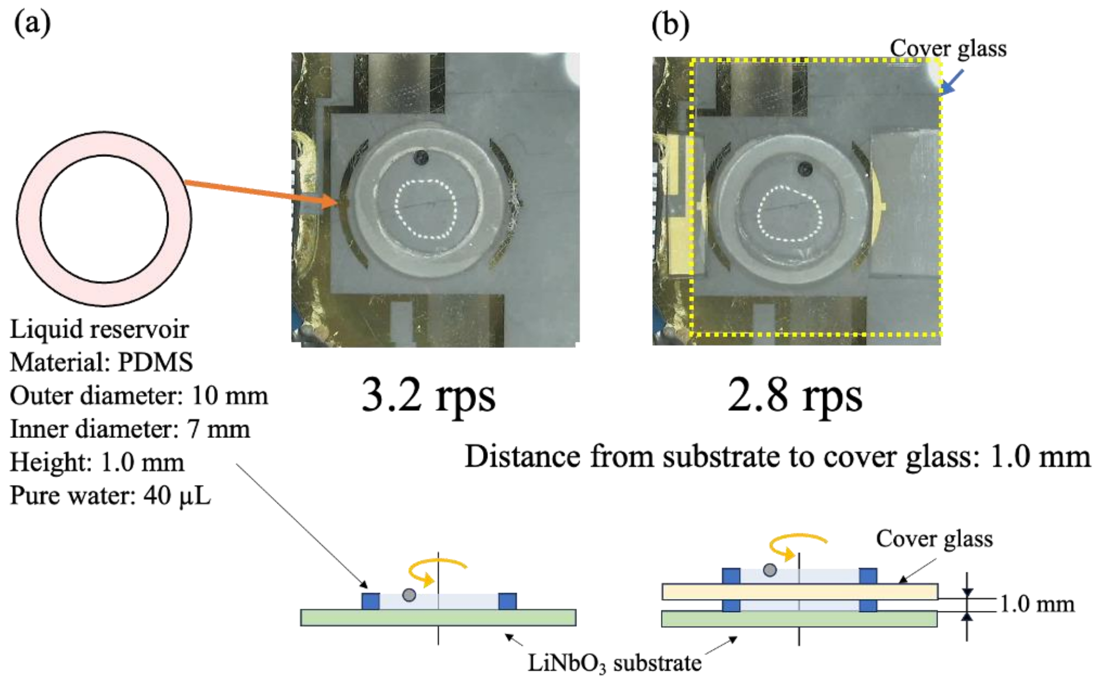

Figure S1 Experimental overview comparing the rotational speed of a ball floating (a) in a liquid reservoir on a piezoelectric substrate and (b) in a liquid reservoir placed on a cover glass coupled with a piezoelectric substrate through the coupling liquid.
